# Toward Chemical Accuracy for Chemi- and Physisorption with an Efficient Density Functional

**DOI:** 10.1021/acs.jpcc.5c08744

**Published:** 2026-02-17

**Authors:** Manish Kothakonda, Abhirup Patra, Ruiqi Zhang, Jinliang Ning, James Furness, Qing Zhao, Jianwei Sun

**Affiliations:** † Department of Physics and Engineering Physics, 5783 Tulane University, New Orleans, Louisiana 70118, United States; ‡ Delaware Energy Institute, 6572 University of Delaware, 221 Academy Street, Newark, Delaware 19716, United States; § Department of Chemical Engineering, 1848 Northeastern University, Boston, Massachusetts 02115, United States

## Abstract

Understanding molecular adsorption on surfaces underpins many problems in chemistry and materials science. Accurately and efficiently describing the adsorption has been a challenging task for first-principles methods as the process can involve both short-range chemical bond formations and long-range physical interactions, e.g., van der Waals (vdW) interaction. Density functional theory presents an appealing choice for modeling adsorption reactions, although calculations with many exchange-correlation density functional approximations struggle to accurately describe both chemical and physical molecular adsorptions. Here, we propose an efficient density functional approximation that is accurate for both chemical and physical adsorption by concurrently optimizing its semilocal component and the long-range vdW correction against the prototypical adsorption CO/Pt(111) and Ar_2_ binding energy curve. The resulting function opens the door to accurate and efficient modeling of general molecular adsorption.

## Graphical abstract

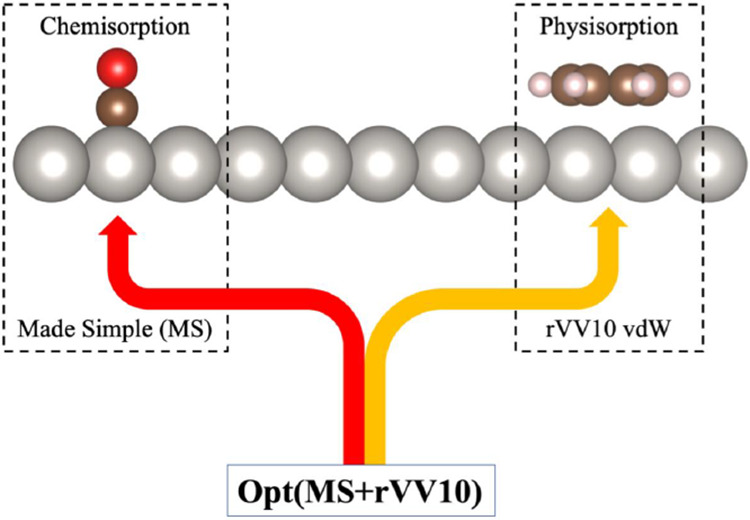

## 1 Introduction

Molecular adsorption on solid surfaces is a challenging yet critical topic in surface science, as a fundamental reaction step for surface chemical reactions. Understanding these processes of molecules adsorbing to solid surfaces is an essential step in understanding important surface phenomena, including semiconductor processing, corrosion, electrochemistry, and heterogeneous catalysis.^1^ In catalysis, adsorption energies are particularly significant because they directly influence reaction rates, selectivity, and stability of intermediates, all of which are essential for efficient catalyst design. Predicting adsorption energies accurately is vital for optimizing catalytic activity, as they determine stable intermediates and accessible transition states and, ultimately, guide the design of active, selective, and durable catalytic materials. Misestimations in adsorption energy can lead to inaccurate predictions, hindering the development of improved catalysts that are essential for industrial and sustainable applications.

However, accurately understanding molecular adsorption on solid surfaces is challenging due to the limitations of available first-principles methods in describing interactions between finite molecules and semi-infinite solid surfaces.^2^ This process can involve the formation of both short-range chemical bonds, termed chemisorption, and long-range physical interactions, e.g., the van der Waals (vdW) interaction, termed physisorption. Calculations using high-level methods such as quantum Monte Carlo (QMC),^3^ coupled-cluster singles and doubles with perturbative triples CCSD­(T),^4^ and random-phase approximation (RPA)^5,6^ can capture both chemisorption and physisorption accurately, but their severe computational cost prevents their routine application to molecular adsorption problems. Calculations using density functional theory (DFT)^7,8^ are comparatively cheap; however, this combination of computational efficiency and useful accuracy has resulted in them becoming a widely accepted workhorse method for surface calculations. The past decades in particular have seen extraordinary progress in DFT-driven computational heterogeneous catalysis,^9,10^ resulting in greatly accelerated computer-based catalyst design.^11–13^ Nevertheless, the limitations of exchange-correlation density functional approximations (DFAs) prevent DFT from reliably predicting both chemisorption and physisorption interactions with equal accuracy, thus posing a significant challenge in catalytic material design.

The accuracy of a DFT calculation is largely determined by the chosen exchange-correlation approximation, many of which have been proposed. Approximate exchange-correlation functionals can broadly be categorized into a hierarchy of increasing sophistication accompanied by increasing computational cost and (ideally) increasing accuracy.^14^ The generalized gradient approximation (GGA) and meta-GGA class of exchange-correlation functionals are well suited for computing surface quantities in chemistry and condensed matter physics since these approximations require only semilocal ingredients and hence remain computationally inexpensive.^15,16^ The absence of nonlocal ingredients means that long-range van der Waals interactions are absent from GGAs and meta-GGAs; however, this can add significant errors to surface calculations.^17–20^ To remedy this, various vdW corrections have been developed to pair with the semilocal approximations.^21–33^ One such case, highly relevant to this work, involved pairing the meta-GGA MS2 ^34,35^ with the rVV10 ^36^ vdW correction without optimizing its parameters. This resulted in a functional that failed to accurately capture the vdW wells, particularly for the well-studied H_2_/Cu­(111) system.^37^ Typically, the vdW correction is fitted independently while the GGA or meta-GGA functional retains its parametrization based on general chemistry applications. This approach often leads to a trade-off: most GGA/meta-GGA functionals with vdW corrections perform well for either chemisorption or physisorption but not both simultaneously. Because the semilocal functional and vdW correction are not designed in conjunction, existing GGA and meta-GGA functionals, even with vdW corrections, struggle to capture both types of adsorption with high accuracy at the same time. Recent advances in many-body dispersion methods, particularly the work by Tkatchenko and colleagues on screened van der Waals interactions and many-body dispersion (MBD) effects,^33,38,39^ have highlighted the importance of collective electronic effects in surface adsorption beyond pairwise additivity. In particular, the comprehensive benchmarking by Maurer et al.^40^ reported mean absolute deviations of 0.06 Å for adsorption heights and 0.16 eV for adsorption energies when comparing DFT + vdW-surf with experiment across a diverse set of adsorption systems, ranging from rare gas atoms to large organic molecules with covalently active subgroups. These benchmarks span both systems dominated by physisorption (e.g., noble gases) and those where chemisorption contributions are also significant.

In this work, we propose a novel density functional with balanced performance for both chemi- and physisorption by concurrently optimizing both semilocal component and the long-range vdW correction against the prototypical CO/Pt(111) and the Ar_2_ binding energy curves; see Section 3.3 for further details. The resulting density functional, termed “Opt­(MS + rVV10)”, which is the topic of this work, is a reparameterization of both the meta-GGA Made Simple (MS) exchange-correlation functional^34^ and the rVV10 long-range vdW correction.^36^ Opt­(MS + rVV10) shows improved and balanced performance for both the physisorption and chemisorption of molecules adsorbed on transition metal surfaces compared to the other DFAs popular for surface science.^41^ Furthermore, Opt­(MS + rVV10) shows the most balanced performance for molecular adsorptions of the DFAs considered and yields both the chemi- and physisorption local minima for graphene adsorbed on a Ni(111) surface, predicting a binding energy curve in close agreement with that from the high-level RPA.^42^

## 2 Methods

The Opt­(MS + rVV10) functional has been implemented in the developmental version of Vienna Ab initio Simulation Package (VASP).^43–45^ All calculations use the pseudopotential project-augmented wave method,^46^ and a high-energy cutoff of 700 eV to truncate the plane-wave basis set. Monkhorst–Pack^47^ *k* meshes for Brillouin zone integration are set to 6 × 6 × 1 for 2 × 2 slabs, and 4 × 4 × 1 for 3 × 3 slabs and Γ point calculation for molecules. Ionic structures were relaxed to a residual force threshold of 0.01 eV/Å per atom, with a total energy tolerance of 10^–5^eV.

Four-layer slabs with the bottom two layers fixed and 15 Å vacuum between the surface species and the next repeated image in the *z*-direction were used for adsorption energies. A dipole correction was applied on the adsorbate atom along the surface normal direction with a magnitude dependent on the charge and distance between the adsorbate and surface atom. Gas-phase molecules used in the calculation of the adsorption energies were optimized in a simulation cell of at least 15 Å vacuum.

To calculate the binding curves for graphene registry with the Ni(111) surface, we put the graphene sheet on top of the surface (as shown in Figure 1 of ref^48^). The metal surface was modeled with a four-layer slab generated with the experimental lattice constants and a 20-Å-thick vacuum layer. The energy cutoff of 600 eV was used, and the Γ-centered 16 × 16 × 1 Monkhorst–Pack^47^ *k* meshes were used.

**Figure 1:**
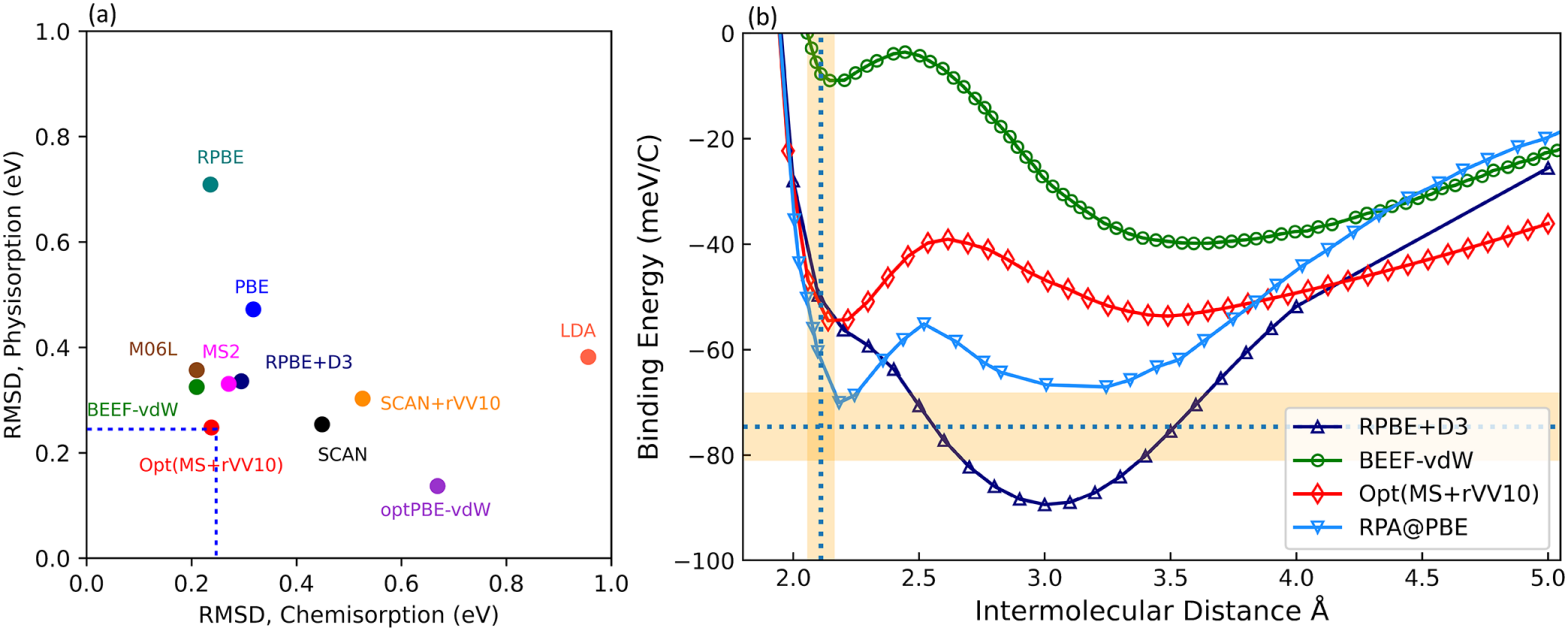
(a) Bivariate plot of the root-mean-square deviation (RMSD) of the chemisorption and physisorption energies per adsorbate for CE39 systems. The blue dashed line represents the 0.25 eV RMSD threshold for both physisorption and chemisorption, with only Opt­(MS + rVV10) edging this boundary. (b) Binding energy curves for graphene adsorbed in Ni(111) surface from RPBE + D3,^51^ BEEF-vdW,^54,55^ and Opt­(MS + rVV10) compared with RPA^42^ results. The blue dotted lines indicate the experimental adsorption distance^56^ of 2.11 ± 0.07 Å, representing the uncertainty range in the measurement, while the orange shaded regions correspond to the experimental adsorption energy range^57,58^ of 74 ± 8.1 meV, reflecting the uncertainty in the reported values.

## 3 Results and Discussion

### 3.1 Bivariate Plot

The CE39 data set, proposed by Wellendorf et al.,^41^ comprises experimental data for 39 well-defined adsorption energies of various gas molecules adsorbed on eight transition metal surfaces. These experimental adsorption energies have been corrected for zero-point vibrational energy (ZPVE) and RT contributions, following the procedure outlined in ref 41, to approximate 0 K enthalpies. This correction enables a consistent and meaningful comparison to static-lattice DFT energies, which are calculated at 0 K without thermal contributions. It includes two subsets: strongly bound chemisorbed systems and weakly bound physisorbed systems, where van der Waals (vdW) interactions dominate. Figure 1(a) presents a bivariate plot contrasting the performance of Opt­(MS + rVV10) against other widely used density functionals, such as the local density approximation (LDA^49^), generalized gradient approximations (PBE^50^ and RPBE^51^), meta-GGAs (SCAN,^52^ MS2,^53^ and M06L), vdW-corrected GGAs (RPBE+D3, BEEF-vdW,^54^ and optPBE-vdW), and vdW-corrected meta-GGAs (SCAN + rVV10). These functionals were selected due to their popularity in surface chemistry (e.g., RPBE) and broader applications (e.g., PBE).

The LDA functional significantly overestimates chemisorption with a root-mean-square deviation (RMSD) around 1.0 eV but predicts physisorption with reasonable accuracy (0.4 eV RMSD). This unexpected accuracy in physisorption likely results from error cancellation, as LDA generally overestimates electron density overlap bonding while failing to account for long-range vdW interactions. PBE, a GGA functional, improves upon LDA for chemisorption, reducing the RMSD from 1.0 to 0.38 eV, while RPBE further lowers it to 0.22 eV. However, both functionals struggle with physisorption, exhibiting RMSDs of 0.46 and 0.71 eV for PBE and RPBE, respectively. More recently, Sharada et al.^59^ extended the CE39 set by adding two additional adsorption reactions, creating the AD41 benchmark, which has been used to assess new density functional approximations such as the empirically fitted meta-GGA MCML.^60^ This functional, while grounded in physical constraints and informed by experimental and quantum chemistry reference data, is empirically optimized to yield improved accuracy for surface- and gas-phase reaction energetics.

When vdW corrections are applied, RPBE + D3 ^61^ significantly enhances RPBE’s performance, delivering a more balanced description of both chemisorption (0.31 eV RMSD) and physisorption (0.35 eV RMSD). The BEEF-vdW functional, developed using a Bayesian approach to fit exchange-correlation parameters for surface chemistry,^54^ achieves an even better RMSD for chemisorption (0.21 eV) compared to RPBE + D3 and a slightly improved physisorption RMSD (0.34 eV). However, this imbalance may be attributed to the fact that 17 chemisorbed systems from the CE39 data set were included in the BEEF-vdW training set. While optPBE-vdW performs best for physisorbed systems among the tested functionals, its overestimation of chemisorption remains unsatisfactory.^62^

At the meta-GGA level, although SCAN^52^ has been highly successful in addressing longstanding issues in condensed matter physics, such as strongly correlated cuprates and phase transitions,^16,63,64^ it overestimates chemisorption with an RMSD of approximately 0.44 eV. This may be due to self-interaction errors, which can lead to an overestimation of charge transfer between molecules and metal surfaces, as evidenced in CO/Pt(111) adsorption studies.^65^ SCAN, however, has demonstrated an ability to capture intermediate vdW interactions,^66^ making it reasonably accurate for physisorption with an RMSD of around 0.30 eV. Another meta-GGA, M06L, performs exceptionally well for chemisorption, with an RMSD of 0.21 eV, but it is significantly less accurate for physisorption (RMSD of 0.37 eV). This is surprising, given that M06L was the first semilocal functional to successfully capture intermediate vdW interactions.^67^ The discrepancy may be due to the highly empirical nature of M06L, which overrepresents molecular systems, leading to an exaggerated emphasis on chemical bonding at the expense of accurately modeling physisorption.

The addition of the rVV10 vdW correction to SCAN (SCAN + rVV10) results in an overestimation of chemisorption relative to SCAN, while also degrading SCAN’s performance in physisorption. This underscores the delicate balance required when a semilocal density functional is paired with a long-range vdW correction.

Even without a vdW correction, MS2 meta-GGA remains one of the most balanced functionals, offering reasonable accuracy across both chemisorption and physisorption. This balance motivated the pairing of MS2 with the rVV10 vdW correction, alongside a reoptimization of the internal parameters, to enhance accuracy while maintaining balanced performance. As demonstrated, the resulting Opt­(MS + rVV10) functional provides the best balanced performance for both chemisorption (RMSD ∼ 0.24 eV) and physisorption (RMSD ∼ 0.26 eV). We also note that Opt­(MS + rVV10) yields a smooth binding energy curve for H_2_/Cu­(111), with a van der Waals minimum whose position lies within experimental uncertainty, though its depth is overestimated^69,70^ (Figure S2, SI).

The adsorption energy errors for chemisorbed and physisorbed systems obtained using RPBE + D3, BEEF-vdW, SCAN + rVV10, and Opt­(MS + rVV10) are compared in Figure 2, with all errors normalized by the number of adsorbates. Systems to the left of the dashed black line are dominated by covalent bonding, whereas those to the right correspond to vdW-interaction-dominated physisorption. Beyond the reaction-resolved comparison shown in the figure, comprehensive error statistics are evaluated across the full CE39 data set, including adsorption energies computed with the PBE, RPBE, RPBE + D3, optPBE-vdW, BEEF-vdW, MS2, SCAN, SCAN + rVV10, and Opt­(MS + rVV10) functionals (Figure S1, SI), with the corresponding adsorption energies and errors summarized in Tables S2 and S3 (SI). Among the functionals examined, Opt­(MS + rVV10) uniquely and consistently exhibits small signed errors for the reactions considered, highlighting its balanced and robust performance across both chemisorption and physisorption regimes. While the predicted equilibrium adsorption distances are largely insensitive to the exchange–correlation functional, significant differences in the signed adsorption energy errors are observed between PBE and Opt­(MS + rVV10) (Figure S3, SI).

**Figure 2:**
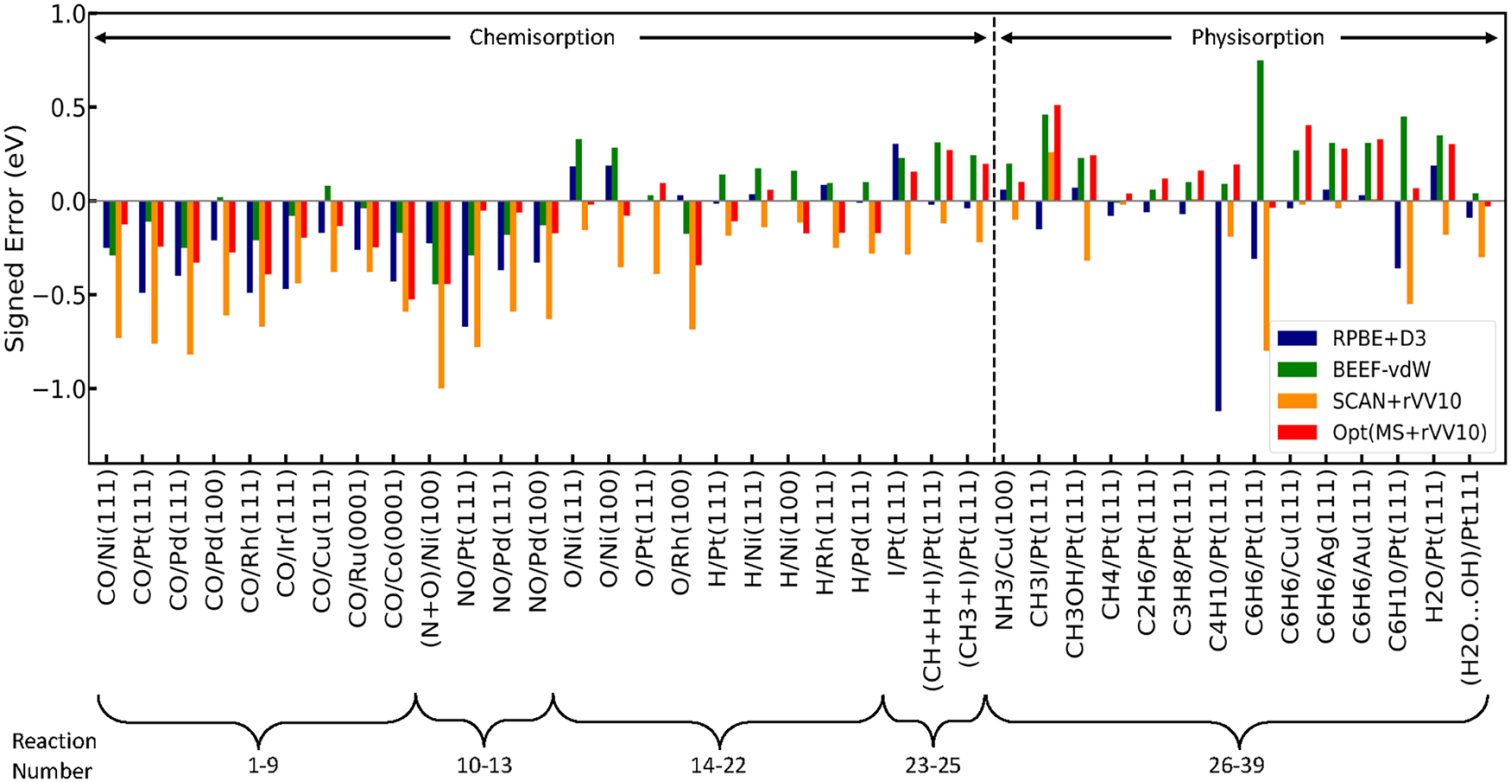
Comparison of errors of chemisorbed and physisorbed systems using RPBE + D3,^61^ BEEF-vdW,^41,54^ SCAN + rVV10,^68^ and Opt­(MS + rVV10). The systems right of the dashed black line are dominated by physisorptions, and those left of the dashed line are dominated by covalent bonding. All errors are scaled by the number of adsorbates.

### 3.2 Graphene Adsorption on Ni(111)

As the CO/Pt(111) and Ar_2_ systems were used to optimize the parameters, we tested the transferability of Opt­(MS + rVV10) for chemisorption and physisorption by applying it to graphene adsorbed on Ni(111). This system was not part of parametrization and is believed to have a challenging double minima of both chemisorption and physisorption in its binding energy curve.

Graphene adsorption on metal is highly metal-dependent, forming strong chemical bonding with some metals and only weak van der Waals interaction with others. Graphene on Ni has shown exceptional electronic properties in semiconducting technology;^71^ however, understanding the interactions between graphene and Ni(111) surface has been challenging for both experiment and theory. A study using angle-resolved photoemission (ARPES) reveals strong chemical bonding combined with weak vdW interactions between the graphene sheet and Ni(111).^72^ Several theoretical studies have been made drawing conflicting conclusions; some predicting dominant chemisorption minima and others predicting deeper physisorption minima.^73–77^ High-level RPA calculations have established that both chemisorption and physisorption minima should have similar depth, in good accordance with the experimentally determined binding energy, as shown in Figure 1(b). Opt­(MS + rVV10) predicts chemisorption and physisorption minima at 2.15 and 3.5 Å, respectively, compared to RPA@PBE values of 2.17 and 3.27 Å. The binding energies for these minima are −58 and −57 meV for Opt­(MS+rVV10), versus −70 and −67 meV for RPA@PBE.

The binding energy curves of graphene adsorbed on Ni surface were calculated using RPBE + D3, BEEF-vdW, and Opt­(MS + rVV10), and are shown in Figure 1(b). These functionals were chosen as representative vdW-corrected functionals that exhibit a good balance between chemisorption and physisorption in Figure 1(a). The binding curves for BEEF-vdW and RPA@PBE are taken from refs 55 and 42, respectively. Low-energy electron diffraction (LEED)^56^ experiments measure the equilibrium separation of the graphene sheet on Ni(111) as 2.11 ± 0.07 Å, which is shown in Figure 1(b) as a vertical dashed blue line with highlighted uncertainty. The corresponding binding energy has been measured by Auger spectroscopy^57,58^ as 74 ± 8.1 meV, illustrated by the horizontal dashed blue line with highlighted uncertainty.

Figure 1(b) shows that RPA@PBE predicts a chemisorption minimum with both the equilibrium position and the binding energy in good agreement with the experimental data. Several studies and RPA@PBE support that graphene on Ni(111) has an additional minimum due to strong chemical and physical adsorption present in the graphene on Ni(111) system.^42,57,58^ BEEF-vdW accurately predicts the chemisorption separation distance, although the binding energy is greatly underestimated. Surprisingly, BEEF-vdW predicts a long-range physisorption minimum that is much deeper than its chemisorption minimum. Furthermore, RPBE + D3 shows a clear physisorption minimum at the expected separation, but the short-range chemisorption minimum is absent. Finally, the newly optimized Opt­(MS + rVV10) functional agrees well by having a double minimum similar to RPA@PBE. Even though the binding energy predicted by the Opt­(MS + rVV10) is slightly higher than the RPA@PBE and experimental uncertainties, the minimum intermolecular distance of chemisorption and physisorption along with the binding energies show the closest agreement to the benchmark and experimental data.

### 3.3 Determination of Parameters for Opt­(MS + rVV10)

We now present the procedure used to determine and optimize the parameters of the Opt­(MS + rVV10) functional. The exchange-correlation energy of Opt­(MS + rVV10) is expressed as

1 $$E_{\mathrm{xc}}^{\mathrm{opt}\left(\mathrm{MS}+\mathrm{rVV}10\right)}=E_{\mathrm{xc}}^{\mathrm{MS}}\left[n;\kappa ,b\right]+E_{c}^{\mathrm{rVV}10}\left[n;\beta \right]$$

Here, κ and *b* are tunable parameters in the MS2 functional, while β is a parameter in the rVV10 functional. In the original formulation of the MS2 meta-GGA, κ = 0.504 and *b* = 4.0 were optimized to fit the atomization energies of six molecules (AE6 set), and the barrier heights of six reactions (BH6 set).^78^ Similarly, in rVV10, β = 6.3 was fitted to the interaction energies of 22 predominant dispersion-dominated complexes (S22 set).^79^

In this work, we reoptimized κ, *b*, and β to improve the functional’s accuracy for both chemisorption and physisorption. Since the MS2 meta-GGA is known to partially capture intermediate-range vdW interactions,^66^ it was necessary to refit β of rVV10 to avoid overestimating vdW forces, which could lead to double-counting. The β parameter of rVV10 was therefore optimized for each (κ, *b*) pair by fitting the binding energy curve of *Ar*_2_, using highly accurate coupled-cluster CCSD­(T) calculations as the reference.^80^ The κ and *b* of MS2 were then reoptimized by fitting to the experimental adsorption energy of CO on Pt(111), a well-studied surface system with reliable experimental data.^81^

We minimized the absolute error in the CO/Pt(111) adsorption energy with respect to κ and *b* using a least-squares fit to a third-order polynomial in the two-dimensional κ–*b* space. This approach allowed us to efficiently explore the parameter space and identify optimal values. The resulting heat map (Figure 3(a)) revealed a well-defined minimum at κ = 0.25 and *b* = 0.91, with β = 26.26; see Table S1, SI. This set of parameters delivered the lowest error for the CO/Pt(111) adsorption energy.

**Figure 3:**
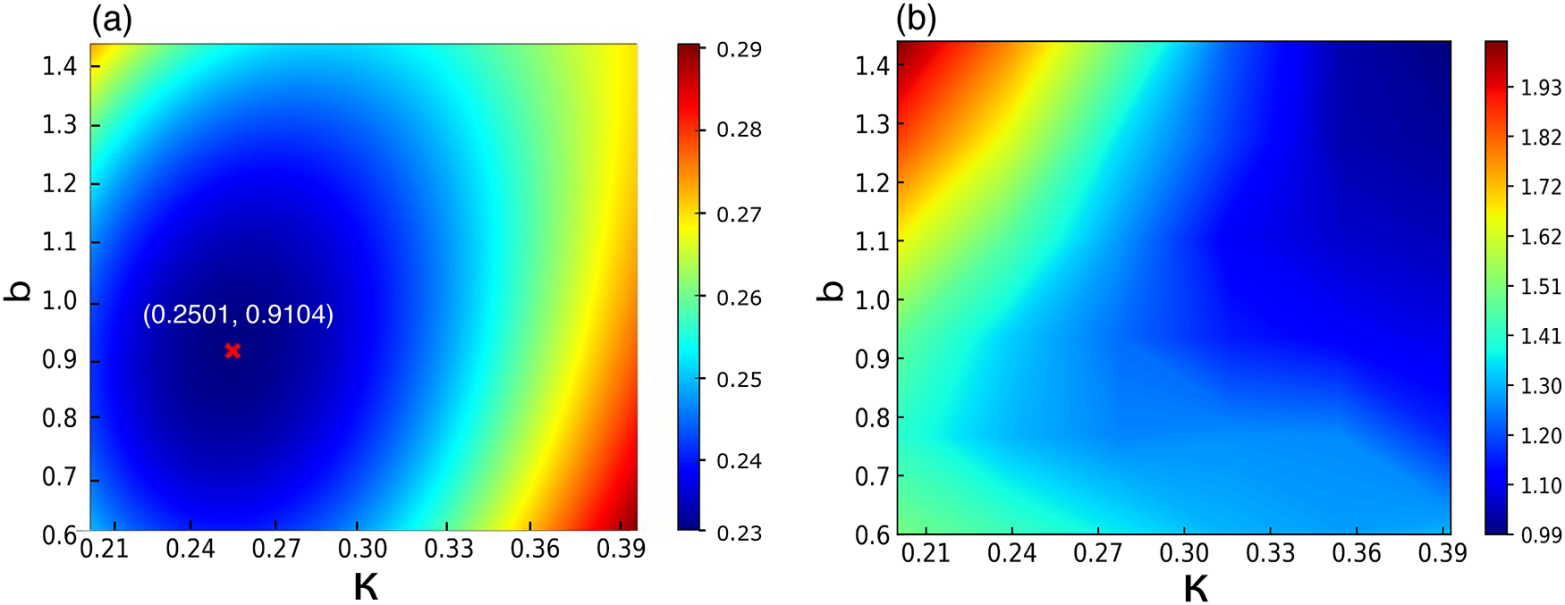
(a) Heat map of third-order polynomial function fitted to the absolute errors of CO adsorbed on the Pt(111) surface as a function of κ and *b* fitting parameters. Errors are in eV. The minimizing parameters are marked in red as κ = 0.2501 and *b* = 0.9104. (b) Heat map of normalized absolute errors of atomization energies subset AE6.^78^

When using AE6 atomization energies as the optimization objective for κ and *b*, a very different minimum was found, located in the upper-right corner of the κ–*b* parameter space (Figure 3(b)). A similar minimum emerged when both the AE6 and BH6 sets were used, in line with the original MS2 parametrization. These findings suggest that developing DFT exchange-correlation functionals for molecular adsorption on solid surfaces requires distinct optimization strategies compared to those used for general chemistry applications.

We conducted additional testing of the newly optimized Opt­(MS + rVV10) functional on a set of small benchmark systems. These tests included the atomization energies from the AE6 set,^78^ hydrogen-transfer barrier heights from the BH6 set,^78^ and lattice constants from the LC20 set,^82^ comprising 20 solids. The performance of Opt­(MS + rVV10) was compared against those of other van der Waals-corrected functionals, including RPBE + D3, BEEF-vdW, and SCAN + rVV10 (the latter being a widely regarded general-purpose functional). The results are summarized in Table 1. For AE6 atomization energies, SCAN + rVV10 and BEEF-vdW showed better accuracy than did Opt­(MS + rVV10), which exhibited slightly higher errors. For BH6 barrier heights, both BEEF-vdW and Opt­(MS + rVV10) performed well, with mean absolute errors (MAEs) of 5.4 and 5.6 kcal/mol, respectively. In the case of LC20 lattice constants, Opt­(MS + rVV10) and SCAN + rVV10 were the most accurate, with MAEs of 0.020 and 0.021 Å, respectively. These results demonstrate that Opt­(MS + rVV10) performs competitively in terms of accuracy, particularly for surface adsorption, while still maintaining reasonable accuracy in other general-purpose tasks.

**Table 1:** Error Statistics for the AE6, BH6, and LC20 Test Sets Using RPBE + D3, BEEF-vdW, SCAN + rVV10, and Opt­(MS + rVV10) Dispersion Corrected Density Functionals

	RPBE + D3	BEEF-vdW	SCAN + rVV10	Opt(MS + rVV10)
Atomization energies (kcal/mol) of the AE6 molecules				
ME	–6.0	–2.8	1.7	–2.5
MAE	8.2	4.3	3.8	5.8
Barrier Heights (kcal/mol) of the BH6 transition states				
ME	–7.6	–5.3	–7.8	–5.6
MAE	7.6	5.4	7.8	5.6
Lattice constants (Å) of the LC20 solids				
ME	0.070	0.064	–0.020	0.008
MAE	0.075	0.071	0.021	0.020

## 4 Conclusion

We present Opt­(MS + rVV10), a novel exchange–correlation van der Waals-corrected functional that represents a significant advancement in modeling molecular adsorption on solid surfaces. This work introduces a new approach in which the semilocal meta-GGA and the long-range vdW correction are simultaneously optimized, ensuring a balanced and physically grounded description of both chemisorption and physisorption. In particular, we demonstrate that the widely used AE6 molecular benchmark is insufficient for chemisorption and that reliable reference adsorption energies, such as CO/Pt(111), are essential for meaningful DFA development. Accordingly, the internal parameters κ and *b* of MS2 and the β parameter of rVV10 were refitted using only two well-understood systems: CO/Pt(111) for chemisorption and Ar_2_ for long-range vdW interactions. Despite this minimal and physically motivated calibration, Opt­(MS + rVV10) exhibits predictive performance on the well-established CE39 adsorption data set, which includes both chemisorbed and physisorbed complexes. Testing on graphene adsorption on Ni(111), a system known for its challenging double minima in the binding energy curve, further shows that Opt­(MS + rVV10) closely aligns with the high-level RPA@PBE benchmark. Additional benchmarking on the AE6, BH6, and LC20 data sets reveals that while Opt­(MS + rVV10) yields slightly lower performance on AE6, it remains competitive for hydrogen-transfer barriers and lattice constants, delivering overall reliable accuracy across diverse systems. While many interesting systems, especially those simultaneously involving covalent bonding and vdW interactions, remain to be tested in future work, the present results already demonstrate clear improvements over existing vdW-corrected DFAs such as BEEF-vdW, RPBE + D3, and SCAN + rVV10. Looking ahead, we anticipate that incorporating many-body vdW contributions into the simultaneous optimization framework with semilocal meta-GGA will further enhance the accuracy of Opt­(MS + rVV10) for molecular adsorption on surfaces, and this will be an important direction for future development. Its balanced treatment of chemisorption and physisorption also makes Opt­(MS + rVV10) well suited for complex catalytic environments, such as zeolites, where strong chemical bonding and weak vdW interactions jointly govern hydrocarbon adsorption and reactivity. Moreover, its ability to capture subtle electronic interactions suggests promising applications to transition metal electrocatalysis, including CO_2_ reduction, where accurate adsorbate binding energies and interfacial charge transfer are essential for predicting activity and selectivity.^83^

## Supplementary Material



## Data Availability

The patch code, data, main results, and the structures to reproduce this work are available at: https://github.com/manishkothakonda/Opt-MS-rVV10-functional

## Notes

Optimization of parameters and minimization of a third-order polynomial function; absolute errors of CO adsorption on Pt(111) with respect to κ, *b*, and β; DFT adsorption energies for systems constituting the CE39 data set; DFT adsorption energy errors for the CE39 data set with overall error statistics; error statistics for chemisorption, physisorption, and the full CE39 data set; H_2_/Cu­(111) van der Waals potential well and schematic illustration; adsorption distances and signed errors as a function of reaction number; CE39 signed errors and bond length comparisons between Opt­(MS + rVV10) and PBE; and references (PDF)

## ∥

(Presently at Shell Technology Center, Houston, TX 77082, United States)
