# Correction to “Toward Chemical Accuracy for Chemi- and Physisorption with an Efficient Density Functional”

**DOI:** 10.1021/acs.jpcc.6c03841

**Published:** 2026-07-28

**Authors:** Manish Kothakonda, Abhirup Patra, Ruiqi Zhang, Jinliang Ning, James Furness, Qing Zhao, Jianwei Sun

**Affiliations:** † Department of Physics and Engineering Physics, 5783 Tulane University, New Orleans, Louisiana 70118, United States; ‡ Delaware Energy Institute, University of Delaware, 221 Academy Street, Newark, Delaware 19716, United States; § Department of Chemical Engineering, 1848 Northeastern University, Boston, Massachusetts 02115, United States

In the original manuscript, the value of parameter *c* was not explicitly provided. We wish to clarify that *c* was refitted to the hydrogen atom exchange energy, using the updated value of κ. With the updated κ = 0.2501, the optimized value of the parameter is *c* = 0.391596228. These values supersede any implicit assumptions about *c* in the original manuscript, and readers are encouraged to use these parameters for reproducing the results presented therein.

We thank Timo Lebeda for bringing this to our attention.

## Data Availability

The patch code, data, main results, and the structures to reproduce this work are available at: https://github.com/manishkothakonda/Opt-MS-rVV10-functionaldata-availability

## ∇

Shell Technology Center, Houston, TX 77082, USA

