# Supplementary material for: Toward Chemical Accuracy for Chemi- and Physisorption with an Efficient Density Functional
Source: J Phys Chem C Nanomater Interfaces. 2026 Feb 17;130(8):2997–3004. doi: 10.1021/acs.jpcc.5c08744 (PMC12951556; doi:10.1021/acs.jpcc.5c08744)
Supplement: Supplementary file 1 [file jp5c08744_si_001.pdf]

Supporting Information for Towards Chemical Accuracy for Chemi- and Physi-Sorption with an Efficient Density Functional

Manish Kothakonda,[1] Abhirup Patra,[2] Ruiqi Zhang,[1] Jinliang Ning,[1] James Furness,[1] Qing Zhao,[3] Jianwei Sun[1*]

[1]Department of Physics and Engineering Physics, Tulane University, New Orleans, LA 70118, USA
[2]Delaware Energy Institute, University of Delaware, 221 Academy Street, Newark, Delaware 19716, United States
(Presently at Shell Technology Center, Houston, TX 77082, United States)
[3]Department of Chemical Engineering, Northeastern University, Boston, MA 02115

*E-mail: jsun@tulane.edu

Contents

1. Section S1: Optimization of parameters and minimization of 3rd order polynomial function S2

2. Table S1: Absolute errors of CO adsorbed on Pt(111) with respect to κ, b and β......................................S3

3. Table S2: DFT Adsorption energies (eV) for systems constituting the CE39 dataset.................................S4

4. Table S3: DFT errors adsorption energies (eV) for systems constituting the CE39 dataset. Overall error statistics (eV) are also reported at the end..S5

5. Figure S2: Error statistics for (a) chemisorption and (b) physisorption (c) CE39 Full data set..................S6

6. Figure S3:(a) H_2/Cu(111) vdW well (b) schematic S7

7. Figure S4: (a) Adsoption distances Vs reaction number (b) Signed error Vs reaction number S8

8. Table S4: CE39 signed errors & bond lengths (Opt(MS+rVV10) vs PBE) S9

9. References..S10

S1. OPTIMIZATION OF PARAMETERS AND MINIMIZATION OF 3^{rd}RDER POLYNOMIAL FUNCTION

The standard semilocal exchange energy of spin-unpolarized density for MS[1–4] is defined as:

$$E_{\mathrm{x}}^{\mathrm{sl}}[n] = \int d^3\mathbf{r}n(\mathbf{r})\varepsilon_{\mathrm{x}}^{\mathrm{unif}}(n)F_{\mathrm{x}}(p,\alpha), \tag{S1}$$

where, $\varepsilon_{\mathrm{x}}^{\mathrm{unif}}(n) = -(3/4\pi^2)(3\pi^2 n)^{1/3}$ is the exchange energy per particle of a uniform electron gas (UEG), $p = |\nabla n|^2/[4(3\pi^2)^{2/3}n^{8/3}] = s^2$ with s to be the reduced density gradient, and F_{x} the enhancement factor with $F_{\mathrm{x}} = 1$ for LSDA. The equation S1 explains the separation of α- and p-dependence[3], in exchange enhancement factor as α is vital in recognizing different orbital-overlapping regions[5], where $\alpha = 0$ is single orbital region which characterizes covalent bonding, $\alpha \approx 1$ identifies the slowly varying density regime characterizing metallic bonding, and $\alpha \geq 1$ is weakly overlapped region for non-covalent interactions. For MS2 $F_x(p,\alpha)$ in equation S1 is given by:

$$F_{\mathrm{x}}^{\mathrm{int}}(p,\alpha) = F_{\mathrm{x}}^{1}(p) + f(\alpha)[F_{\mathrm{x}}^{0}(p) - F_{\mathrm{x}}^{1}(p)] \tag{S2}$$

where,

$$F_{\mathrm{x}}^{1}(p) = F_{\mathrm{x}}^{\mathrm{int}}(p,\alpha = 1) = 1 + \kappa - \frac{\kappa}{1 + \frac{\mu^{\mathrm{GE}}p}{\kappa}}, \tag{S3}$$

and

$$F_{\mathrm{x}}^{0}(p) = F_{\mathrm{x}}^{\mathrm{int}}(p,\alpha = 0) = 1 + \kappa - \frac{\kappa}{1 + \frac{\mu^{\mathrm{GE}}p+c}{\kappa}}, \tag{S4}$$

is the single orbital enhancement, and

$$f(\alpha) = \frac{(1-\alpha^2)^3}{1+\alpha^3+b\alpha^6}, \tag{S5}$$

is a function that interpolates and extrapolates these limits. In Eq. S5 the parameter b controls the α which identifies the different types of bonding regions namely, slowly varying density, metalic bonding and weak non-covalnet interactions. In addition, b parameter controls the interpolation behaviour and sets the $\alpha \to \infty$ limit. In Eqs. S3 and S4, κ controls the functional's density gradient response through s.

Now we discuss about the the rVV10 non-local correlation functional[6] which is a modification of the VV10 correlation kernel[7] which takes the form based on Rutgers-Chalmers vdW-DFs shown in the Eq. S6.

$$E_{\mathrm{c}}^{\mathrm{nl}} = \frac{1}{2}\int\int d\mathbf{r}n(\mathbf{r})\left[d\mathbf{r}'\phi(\mathbf{r},\mathbf{r}')n(\mathbf{r}') + \beta\right] \tag{S6}$$

where $n(\mathbf{r})$ is the electron density and $\phi(\mathbf{r},\mathbf{r}')$ is the kernel describing the density-density interactions. β controls short range damping of R^{-6} (R=$|r-r'|$) asymptote. To ensure zero $E_{\mathrm{c}}^{\mathrm{nl}}$ for the uniform electron gas, $\beta = \frac{1}{32}(3/b_{\mathrm{vdW}})^{\frac{3}{4}}$ in Hartrees is required for rVV10. bparam in the original form is changed to b_{vdW} to avoid confusion with b in mGGA-MS. The two empirical parameters C and b_{vdW} appear in the kernel: C =0.0093 is chosen for accurate $-C_6/R^6$ vdW interaction between molecules at large separation R, and b_{vdW} which is dependent on β controlling the damping of $E_{\mathrm{c}}^{\mathrm{nl}}$ is fitted to the Ar_2 dimer binding energy curve for each κ and b.

In the original form in MS2, b = 4.0, and κ = 0.504 was determined by Averaged Mean Absolute Errors of atomization energies subset AE6, and barrier heights subset BH6 minimized by surface fitted function. Here, we combine the two parameters of MS (b, κ) and one parameter of rVV10 (b_{vdW}) fitted together to the Ar_2 dimer binding energies and adsorption energy of CO/Pt(111). A coarse grid in κ and b two dimensional fitting space is made. The b_{vdW} was re-fit for each (κ, b) for six points of Ar_2 binding curve. Then we calculate adsorption energies of CO/Pt(111) surface for each of (κ, b). The absolute errors of CO/Pt(111) adsorption shown in Table S1 was fitted to 3rd order polynomial equation in κ and b 2D space is represented in the figure 3 in the main manuscript was minimized to optimize the parameters $\kappa = 0.2501$, b = 0.9104 and $\beta = 26.26$.

TABLE S1. Absolute errors of CO adsorbed on Pt(111) with respect to κ, b and β

**Kappa (κ)**	**b**	**c**	β	**Absolute Error CO/Pt(111) eV**
0.2	0.6	0.8650485	13.6	0.2508110
0.2	0.8	0.8650485	24.51	0.2435690
0.2	1	0.8650485	72.85	0.2346810
0.2	1.2	0.8650485	81.46	0.2611830
0.2	1.44	0.8650485	288.33	0.2746470
0.245	0.6	0.41234953	12.63	0.2382290
0.245	0.8	0.41234953	20.38	0.2335860
0.245	1	0.41234953	37.1	0.2375960
0.245	1.2	0.41234953	98.84	0.2290010
0.245	1.44	0.41234953	138.2	0.2480500
0.29	0.6	0.28790598	10.72	0.2456092
0.29	0.8	0.28790598	15.5	0.2404080
0.29	1	0.28790598	21.91	0.2389270
0.29	1.2	0.28790598	31.8	0.2409970
0.29	1.44	0.28790598	60.73	0.2629710
0.34	0.6	0.22454422	8.91	0.2639880
0.34	0.8	0.22454422	12.08	0.2536260
0.34	1	0.22454422	15.09	0.2542321
0.34	1.2	0.22454422	18.7	0.2528130
0.34	1.44	0.22454422	23.64	0.2508810
0.3928	0.6	0.18741979	7.45	0.2926470
0.3928	0.8	0.18741979	9.47	0.2796840
0.3928	1	0.18741979	11.38	0.2746390
0.3928	1.2	0.18741979	13.2	0.2723210
0.3928	1.44	0.18741979	15.36	0.2713650

TABLE S2. DFT Adsorption energies (eV) for systems constituting the CE39 dataset.

**Reaction Name**	**Site**	**C**	**Expt**	**LDA**	**PBE**	**RPBE**	**RPBE+D3**	**optPBE-vdW**	**BEEF+vdW**	**MS2**	**SCAN**	**SCAN+rVV10**	**Opt(MS+rVV10)**
CO/Ni(111)	fcc	1/4	-1.28	-2.7872	-1.81	-1.42	-1.530	-1.93	-1.57	-1.49	-1.89	-2.01	-1.40
CO/Pt(111)	top	1/4	-1.28	-2.2152	-1.64	-1.36	-1.770	-1.68	-1.39	-1.49	-1.94	-2.04	-1.52
CO/Pd(111)	fcc	1/4	-1.49	-2.7872	-1.95	-1.58	-1.890	-2.08	-1.74	-1.95	-2.19	-2.31	-1.82
CO/Pd(100)	bridge	1/4	-1.63	-2.652	-1.86	-1.52	-1.840	-1.94	-1.61	-1.86	-2.09	-2.24	-1.90
CO/Rh(111)	top	1/4	-1.47	-2.5376	-1.84	-1.57	-1.960	-1.96	-1.68	-1.82	-2.04	-2.14	-1.86
CO/Ir(111)	top	1/4	-1.7	-2.5376	-1.93	-1.67	-2.170	-2.03	-1.78	-1.84	-2.04	-2.14	-1.90
CO/Cu(111)	top	1/4	-0.59	-1.3728	-0.75	-0.48	-0.760	-0.78	-0.51	-0.67	-0.91	-0.97	-0.72
CO/Ru(0001)	top	1/4	-1.67	-2.496	-1.88	-1.61	-1.930	-1.96	-1.71	-1.82	-1.95	-2.05	-1.92
CO/Co(0001)	top	1/4	-1.23	-2.4336	-1.63	-1.33	-1.660	-1.71	-1.4	-1.32	-1.72	-1.82	-1.76
(N+O)/Ni(100)	hollow	1/4	-3.1	-6.0112	-4.37	-3.72	-3.550	-5.27	-3.99	-4.27	-4.94	-5.1	-3.99
NO/Pt(111)	hollow	1/4	-1.23	-2.8704	-1.83	-1.43	-1.900	-2.23	-1.52	-1.46	-1.91	-2.01	-1.28
NO/Pd(111)	fcc	1/4	-1.89	-3.276	-2.23	-1.83	-2.260	-2.8	-2.07	-2.18	-2.37	-2.48	-1.95
NO/Pd(100)	fcc	1/4	-1.69	-3.2344	-2.05	-1.63	-2.020	-2.6	-1.82	-2.02	-2.19	-2.32	-1.86
O/Ni(111)	hollow	1/4	-5.03	-5.8864	-4.58	-4.08	-4.660	-6.12	-4.37	-4.48	-5.24	-5.34	-5.07
O/Ni(100)	fcc	1/4	-5.49	-6.6664	-5.07	-4.5	-5.110	-6.66	-4.92	-5.18	-6.19	-6.2	-5.65
O/Pt(111)	fcc	1/9	-2.16	-3.2552	-2.37	-1.86	-2.170	-3.2	-2.1	-2.43	-2.91	-2.94	-1.97
O/Rh(100)	hollow	1/4	-3.68	-5.7408	-4.33	-3.76	-3.620	-5.79	-4.03	-4.82	-4.92	-5.05	-4.37
H/Pt(111)	top	1/4	-0.75	-1.664	-0.96	-0.65	-0.780	-0.79	-0.47	-0.86	-1.11	-1.12	-0.97
H/Ni(111)	fcc	1/4	-1.04	-1.9136	-1.1	-0.77	-0.970	-1.05	-0.69	-1.28	-1.28	-1.32	-0.92
H/Ni(100)	hollow	1/4	-0.9	-1.8928	-1.01	-0.65	-0.910	-0.9	-0.58	-1.19	-1.29	-1.13	-1.25
H/Rh(111)	fcc	1/4	-0.75	-1.872	-1.07	-0.76	-0.580	-0.91	-0.56	-1.2	-1.22	-1.25	-1.09
H/Pd(111)	fcc	1/4	-0.93	-1.9136	-1.2	-0.88	-0.950	-1.1	-0.73	-1.4	-1.44	-1.49	-1.27
I/Pt(111)	fcc	1/4	-3.24	-3.9104	-2.84	-2.27	-2.630	-3.28	-2.78	-2.99	-3.49	-3.81	-2.93
(CH+H+I)/Pt(111)	fcc	1/4	-4.72	-5.4184	-3.85	-3.05	-4.800	-4.27	-3.47	-3.7	-4.88	-5.2	-3.63
(CH3+I)/Pt(111)	top	1/4	-2.17	-2.496	-1.66	-1.22	-2.250	2.12	-1.68	-1.76	-2.36	-2.61	-1.77
NH3/Cu(100)	top	1/4	-0.59	-0.9048	-0.43	-0.21	-0.530	-0.6	-0.39	-0.46	-0.58	-0.69	-0.49
CH3I/Pt(111)	fcc	1/4	-0.87	-0.7904	-0.25	0	-1.020	-0.71	-0.41	-0.34	-0.39	-0.61	-0.36
CH3OH/Pt(111)	top	1/4	-0.57	-0.728	-0.21	-0.05	-0.500	-0.64	-0.34	-0.33	-0.73	-0.89	-0.33
CH4/Pt(111)	fcc	1/4	-0.14	-0.2184	-0.02	-0.01	-0.220	-0.21	-0.15	-0.04	-0.08	-0.16	-0.10
C2H6/Pt(111)	fcc	1/9	-0.28	-0.4472	-0.04	-0.02	-0.340	-0.35	-0.22	-0.07	-0.14	-0.27	-0.16
C3H8/Pt(111)	top	1/9	-0.4	-0.6552	-0.05	-0.01	-0.470	-0.48	-0.3	-0.1	-0.2	-0.39	-0.24
C4H10/Pt(111)	top	1/9	-0.5	-0.8424	-0.07	-0.02	-1.620	-0.62	-0.41	-0.13	-0.27	-0.69	-0.30
C6H6/Pt(111)	bridge	1/9	-1.68	-2.756	-0.98	-0.17	-1.990	-1.8	-0.93	-1.59	-1.94	-2.48	-1.72
C6H6/Cu(111)	fcc	1/9	-0.68	-0.8008	-0.05	0	-0.720	-0.74	-0.41	-0.27	-0.3	-0.7	-0.28
C6H6/Ag(111)	fcc	1/9	-0.63	-0.6344	-0.05	0	-0.570	-0.67	-0.32	-0.22	-0.27	-0.67	-0.35
C6H6/Au(111)	fcc	1/9	-0.72	-0.4056	-0.05	-0.01	-0.690	-0.76	-0.41	-0.26	-0.38	-0.71	-0.39
C6H10/Pt(111)	top	1/9	-1.26	-1.8408	-0.63	-0.07	-1.620	-1.55	-0.81	-0.95	-1.38	-1.81	-1.19
H2O/Pt(111)	top	1/4	-0.57	-0.6552	-0.21	-0.05	-0.380	-0.39	-0.22	-0.09	-0.66	-0.75	-0.27
(H2O...OH)/Pt111	hex	1/9	-0.69	-1.2688	-0.66	-0.19	-0.870	-1.2	-0.61	-0.91	-1.13	-1.29	-0.75

TABLE S3. DFT errors in adsorption energies (eV) for systems constituting the CE39 dataset. Overall error statistics (eV) are also reported at the end

**Reaction Name**	**LDA**	**PBE**	**RPBE**	**RPBE+D3**	**optPBE-vdW**	**BEEF+vdW**	**MS2**	**SCAN**	**SCAN+rVV10**	**Opt(MS+rVV10)**
CO/Ni(111)	-1.507	-0.530	-0.140	-0.250	-0.650	-0.290	-0.210	-0.610	-0.730	-0.12
CO/Pt(111)	-0.935	-0.360	-0.080	-0.490	-0.400	-0.110	-0.210	-0.660	-0.760	-0.24
CO/Pd(111)	-1.297	-0.460	-0.090	-0.400	-0.590	-0.250	-0.460	-0.700	-0.820	-0.33
CO/Pd(100)	-1.022	-0.230	0.110	-0.210	-0.310	0.020	-0.230	-0.460	-0.610	-0.27
CO/Rh(111)	-1.068	-0.370	-0.100	-0.490	-0.490	-0.210	-0.350	-0.570	-0.670	-0.39
CO/Ir(111)	-0.838	-0.230	0.030	-0.470	-0.330	-0.080	-0.140	-0.340	-0.440	-0.20
CO/Cu(111)	-0.783	-0.160	0.110	-0.170	-0.190	0.080	-0.080	-0.320	-0.380	-0.13
CO/Ru(0001)	-0.826	-0.210	0.060	-0.260	-0.290	-0.040	-0.150	-0.280	-0.380	-0.25
CO/Co(0001)	-1.204	-0.400	-0.100	-0.430	-0.480	-0.170	-0.090	-0.490	-0.590	-0.53
(N+O)/Ni(100)	-2.911	-1.270	-0.620	-0.450	-2.170	-0.890	-1.170	-1.840	-2.000	-0.89
NO/Pt(111)	-1.640	-0.600	-0.200	-0.670	-1.000	-0.290	-0.230	-0.680	-0.780	-0.05
NO/Pd(111)	-1.386	-0.340	0.060	-0.370	-0.910	-0.180	-0.290	-0.480	-0.590	-0.06
NO/Pd(100)	-1.544	-0.360	0.060	-0.330	-0.910	-0.130	-0.330	-0.500	-0.630	-0.17
O/Ni(111)	-0.856	0.450	0.950	0.370	-1.090	0.660	0.550	-0.210	-0.310	-0.04
O/Ni(100)	-1.176	0.420	0.990	0.380	-1.170	0.570	0.310	-0.700	-0.710	-0.16
O/Pt(111)	-1.095	-0.210	0.300	-0.010	-1.040	0.060	-0.270	-0.750	-0.780	0.19
O/Rh(100)	-2.061	-0.650	-0.080	0.060	-2.110	-0.350	-1.140	-1.240	-1.370	-0.69
H/Pt(111)	-0.914	-0.210	0.100	-0.030	-0.040	0.280	-0.110	-0.360	-0.370	-0.22
H/Ni(111)	-0.874	-0.060	0.270	0.070	-0.010	0.350	-0.240	-0.240	-0.280	0.12
H/Ni(100)	-0.993	-0.110	0.250	-0.010	0.000	0.320	-0.290	-0.390	-0.230	-0.35
H/Rh(111)	-1.122	-0.320	-0.010	0.170	-0.160	0.190	-0.450	-0.470	-0.500	-0.34
H/Pd(111)	-0.984	-0.270	0.050	-0.020	-0.170	0.200	-0.470	-0.510	-0.560	-0.34
I/Pt(111)	-0.670	0.400	0.970	0.610	-0.040	0.460	0.250	-0.250	-0.570	0.31
(CH+H+I)/Pt(111)	-0.698	0.870	1.670	-0.080	0.450	1.250	1.020	-0.160	-0.480	1.09
(CH3+I)/Pt(111)	-0.326	0.510	0.950	-0.080	4.290	0.490	0.410	-0.190	-0.440	0.40
NH3/Cu(100)	-0.315	0.160	0.380	0.060	-0.010	0.200	0.130	0.010	-0.100	0.10
CH3I/Pt(111)	0.080	0.620	0.870	-0.150	0.160	0.460	0.530	0.480	0.260	0.51
CH3OH/Pt(111)	-0.158	0.360	0.520	0.070	-0.070	0.230	0.240	-0.160	-0.320	0.24
CH4/Pt(111)	-0.078	0.120	0.130	-0.080	-0.070	-0.010	0.100	0.060	-0.020	0.04
C2H6/Pt(111)	-0.167	0.240	0.260	-0.060	-0.070	0.060	0.210	0.140	0.010	0.12
C3H8/Pt(111)	-0.255	0.350	0.390	-0.070	-0.080	0.100	0.300	0.200	0.010	0.16
C4H10/Pt(111)	-0.342	0.430	0.480	-1.120	-0.120	0.090	0.370	0.230	-0.190	0.20
C6H6/Pt(111)	-1.076	0.700	1.510	-0.310	-0.120	0.750	0.090	-0.260	-0.800	-0.04
C6H6/Cu(111)	-0.121	0.630	0.680	-0.040	-0.060	0.270	0.410	0.380	-0.020	0.40
C6H6/Ag(111)	-0.004	0.580	0.630	0.060	-0.040	0.310	0.410	0.360	-0.040	0.28
C6H6/Au(111)	0.314	0.670	0.710	0.030	-0.040	0.310	0.460	0.340	0.010	0.33
C6H10/Pt(111)	-0.581	0.630	1.190	-0.360	-0.290	0.450	0.310	-0.120	-0.550	0.07
H2O/Pt(111)	-0.085	0.360	0.520	0.190	0.180	0.350	0.480	-0.090	-0.180	0.30
(H2O...OH)/Pt111	-0.579	0.030	0.500	-0.180	-0.510	0.080	-0.220	-0.440	-0.600	-0.06
Mean signed deviation (MSD	-0.823	0.030	0.366	-0.142	-0.281	0.143	-0.014	-0.315	-0.475	-0.026
Mean absolute deviation (MAD	0.843	0.407	0.439	0.243	0.541	0.297	0.352	0.427	0.489	0.275
Root mean square deviation (RMSD)(All)	1.033	0.472	0.610	0.338	0.965	0.390	0.434	0.539	0.620	0.353
Root mean square deviation (RMSD)(1-25)	0.956	0.318	0.236	0.295	0.699	0.210	0.271	0.449	0.526	0.238
Root mean square deviation (RMSD)(26-39)	0.382	0.472	0.709	0.336	0.137	0.325	0.331	0.254	0.303	0.248
Root mean square deviation (RMSD(1-9)	1.077	0.348	0.095	0.372	0.437	0.165	0.243	0.513	0.617	0.298
Root mean square deviation (RMSD(10-13)	1.510	0.502	0.189	0.432	0.979	0.288	0.383	0.669	0.767	0.241
Root mean square deviation (RMSD(14-22)	0.586	0.173	0.243	0.094	0.476	0.188	0.255	0.310	0.330	0.162
Root mean square deviation (RMSD(14-22)	0.238	0.225	0.460	0.178	1.240	0.265	0.202	0.094	0.219	0.214

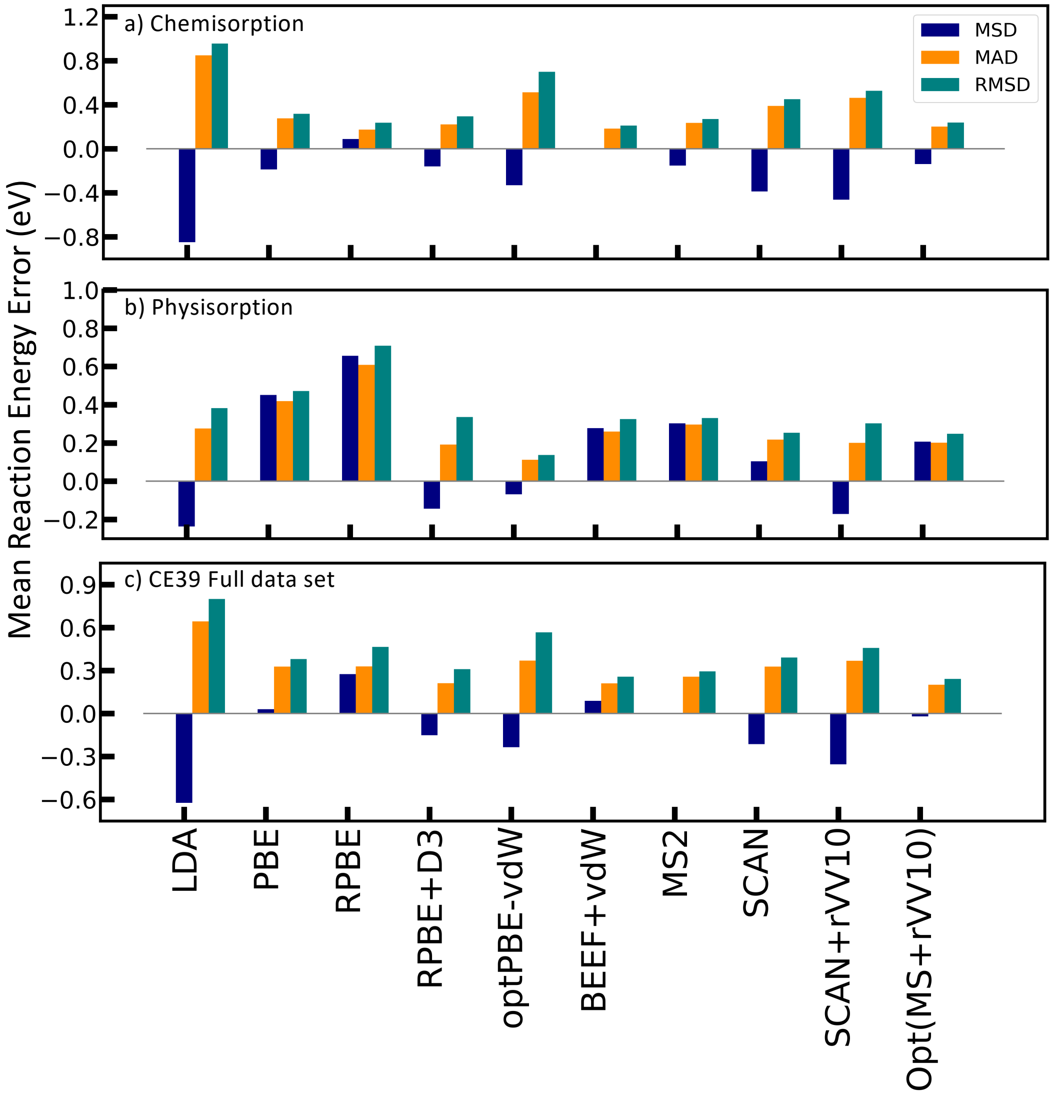

FIG. S1. Error statistics for (a) chemisorption and (b) physisorption (c) CE39 Full data set energies of PBE[8], RPBE[8], RPBE+D3[9], optPBE-vdW[10], BEEF-vdW[8,11], MS2, SCAN, SCAN+rVV10 and Opt(MS+rVV10) functionals with the VASP code[12–14]

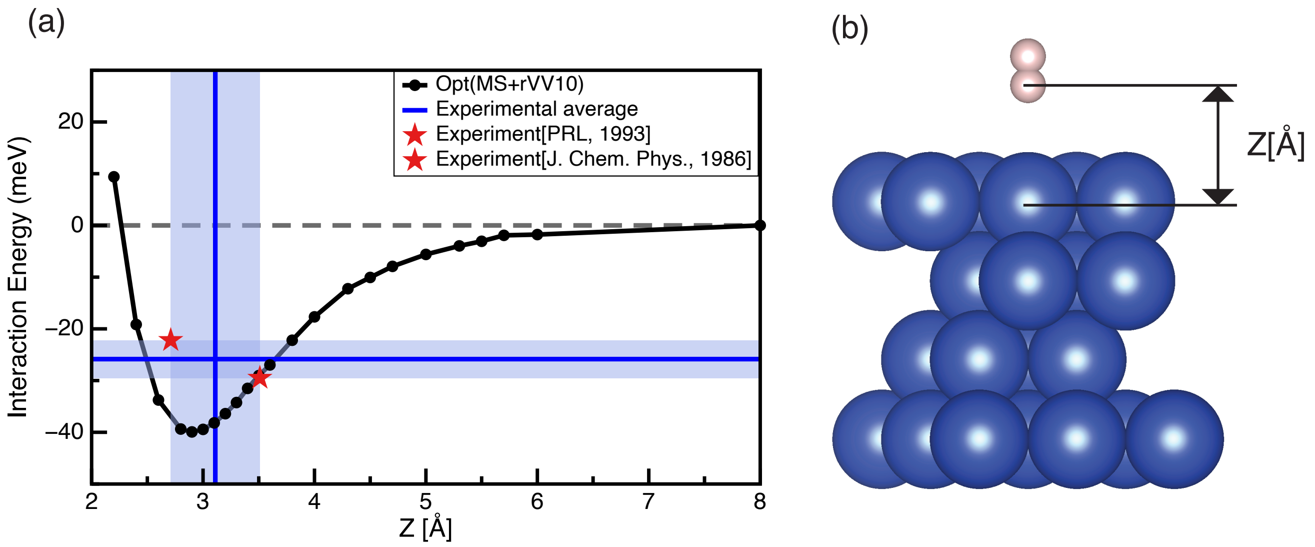

FIG. S2. vdW adsorption well for H_2 on Cu(111). The experimental mean (blue line), averaged from two measurements[15,16], is shown with its uncertainty (blue shaded band). (a) vdW well vs. molecule–surface separation; (b) schematic of H_2 on Cu(111).

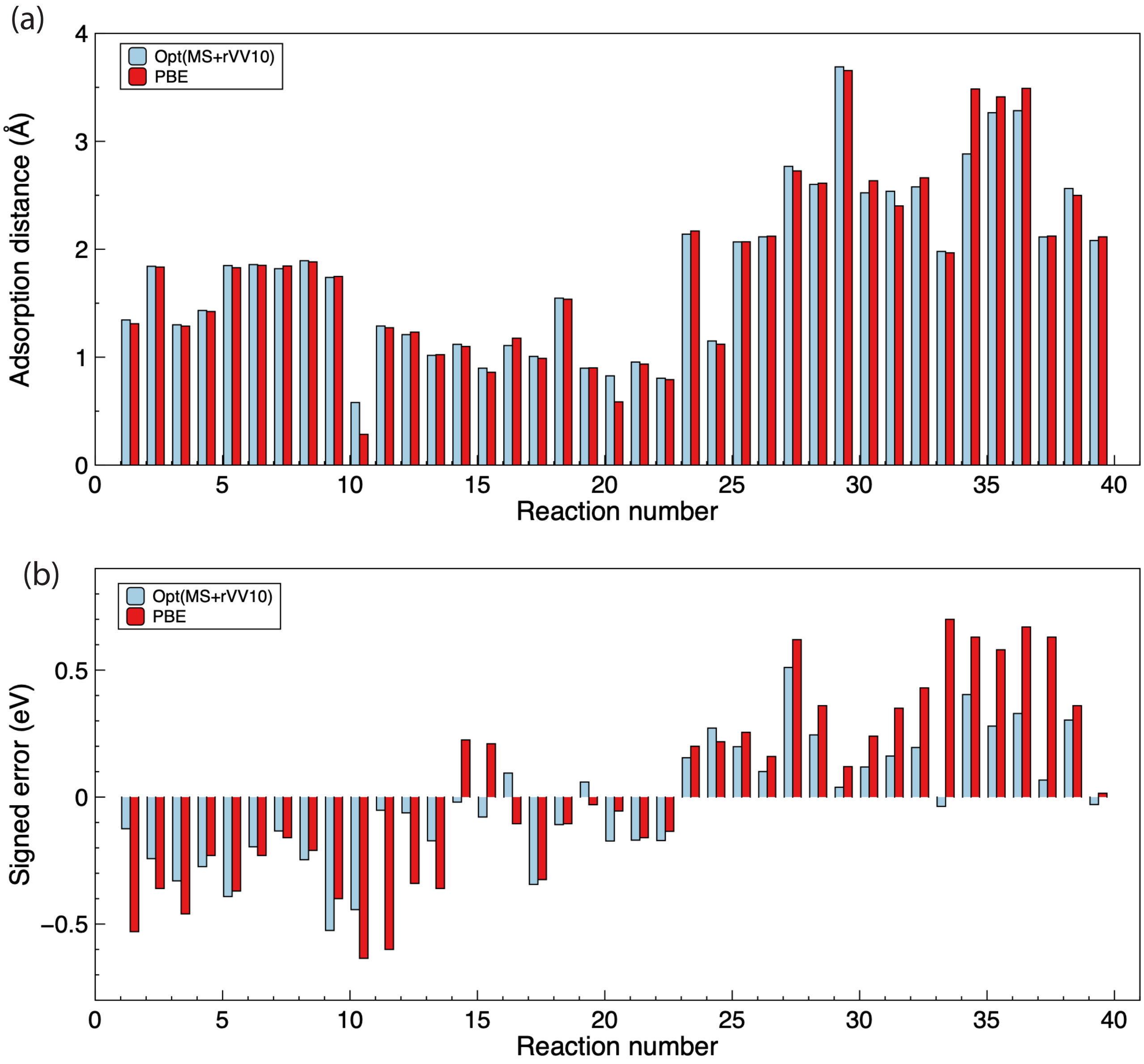

FIG. S3. (a) Optimized equilibrium adsorption distances predicted by Opt(MS+rVV10) and PBE for the CE39 benchmark set, plotted against reaction number. (b) Signed error in adsorption energies, relative to experimental reference values, as a function of reaction number.

TABLE S4. Signed energy errors (Opt(MS+rVV10) vs. PBE) and corresponding bond lengths for CE39 adsorbate–surface systems.

**System**	**Opt(MS+rVV10) signed error (eV)**	**PBE signed error (eV)**	**Opt(MS+rVV10) Bond length (Å)**	**PBE Bond length (Å)**
CO/Ni(111)	-0.1247	-0.53	1.345	1.310
CO/Pt(111)	-0.2425	-0.36	1.842	1.835
CO/Pd(111)	-0.3299	-0.46	1.300	1.288
CO/Pd(100)	-0.2739	-0.23	1.433	1.423
CO/Rh(111)	-0.3917	-0.37	1.849	1.829
CO/Ir(111)	-0.1958	-0.23	1.858	1.851
CO/Cu(111)	-0.1335	-0.16	1.820	1.845
CO/Ru(0001)	-0.2467	-0.21	1.894	1.883
CO/Co(0001)	-0.5252	-0.40	1.739	1.748
(N+O)/Ni(100)	-0.4433	-0.635	0.580	0.285
NO/Pt(111)	-0.0518	-0.60	1.289	1.273
NO/Pd(111)	-0.0622	-0.34	1.209	1.232
NO/Pd(100)	-0.1722	-0.36	1.017	1.023
O/Ni(111)	-0.0198	0.225	1.119	1.099
O/Ni(100)	-0.0785	0.210	0.898	0.860
O/Pt(111)	0.0946	-0.105	1.108	1.176
O/Rh(100)	-0.3439	-0.325	1.007	0.989
H/Pt(111)	-0.1084	-0.105	1.547	1.537
H/Ni(111)	0.0592	-0.030	0.898	0.901
H/Ni(100)	-0.1730	-0.055	0.827	0.586
H/Rh(111)	-0.1696	-0.160	0.955	0.936
H/Pd(111)	-0.1712	-0.135	0.805	0.792
I/Pt(111)	0.1549	0.200	2.140	2.169
(CH+H+I)/Pt(111)	0.2717	0.218	1.150	1.120
(CH3+I)/Pt(111)	0.1985	0.255	2.068	2.069
NH3/Cu(100)	0.1006	0.160	2.115	2.121
CH3I/Pt(111)	0.5104	0.620	2.768	2.726
CH3OH/Pt(111)	0.2448	0.360	2.601	2.612
CH4/Pt(111)	0.0387	0.120	3.690	3.656
C2H6/Pt(111)	0.1186	0.240	2.523	2.635
C3H8/Pt(111)	0.1616	0.350	2.537	2.402
C4H10/Pt(111)	0.1953	0.430	2.578	2.662
C6H6/Pt(111)	-0.0367	0.700	1.980	1.967
C6H6/Cu(111)	0.4038	0.630	2.883	3.485
C6H6/Ag(111)	0.2795	0.580	3.265	3.412
C6H6/Au(111)	0.3292	0.670	3.284	3.491
C6H10/Pt(111)	0.0670	0.630	2.114	2.122
H2O/Pt(111)	0.3034	0.360	2.563	2.499
(H2O...OH)/Pt(111)	-0.0293	0.015	2.081	2.115

[1] J. Sun, B. Xiao, and A. Ruzsinszky, The Journal of Chemical Physics **137**, 051101 (2012).
[2] J. Sun, R. Haunschild, B. Xiao, I. W. Bulik, G. E. Scuseria, and J. P. Perdew, The Journal of chemical physics **138**, 044113 (2013).
[3] J. P. Perdew and Y. Wang, Phys. Rev. B **45**, 13244 (1992).
[4] J. Sun, J. P. Perdew, and M. Seidl, Phys. Rev. B **81**, 085123 (2010).
[5] J. Sun, B. Xiao, Y. Fang, R. Haunschild, P. Hao, A. Ruzsinszky, G. I. Csonka, G. E. Scuseria, and J. P. Perdew, Physical review letters **111**, 106401 (2013).
[6] R. Sabatini, T. Gorni, and S. de Gironcoli, Phys. Rev. B **87**, 041108 (2013).
[7] O. A. Vydrov and T. Van Voorhis, The Journal of Chemical Physics **133**, 244103 (2010).
[8] J. Wellendorff, T. L. Silbaugh, D. Garcia-Pintos, J. K. Nørskov, T. Bligaard, F. Studt, and C. T. Campbell, Surface Science **640**, 36 (2015).
[9] D. Mahlberg, S. Sakong, K. Forster-Tonigold, and A. Groß, Journal of Chemical Theory and Computation **15**, 3250 (2019), pMID: 30964999.
[10] A. J. Hensley, K. Ghale, C. Rieg, T. Dang, E. Anderst, F. Studt, C. T. Campbell, J.-S. McEwen, and Y. Xu, The Journal of Physical Chemistry C **121**, 4937 (2017).
[11] J. Wellendorff, K. T. Lundgaard, A. Møgelhøj, V. Petzold, D. D. Landis, J. K. Nørskov, T. Bligaard, and K. W. Jacobsen, Physical Review B **85**, 235149 (2012).
[12] G. Kresse and J. Hafner, Phys. Rev. B **49**, 14251 (1994).
[13] G. Kresse and J. Furthmüller, Phys. Rev. B **54**, 11169 (1996).
[14] G. Kresse and D. Joubert, Phys. Rev. B **59**, 1758 (1999).
[15] S. Andersson and M. Persson, Physical review letters **70**, 202 (1993).
[16] U. Harten, J. Toennies, and C. Wöll, The Journal of chemical physics **85**, 2249 (1986).
